# Supplementary material for: Pulse Spray Drying for Bovine Skimmed Milk Powder Production
Source: Foods. 2024 Mar 13;13(6):869. doi: 10.3390/foods13060869 (PMC10969098; doi:10.3390/foods13060869)
Supplement: Supplementary file 1 [file foods-13-00869-s001.zip › foods-2905431-supplementary.pdf]

Table S1. Raw values of all the studied parameters on skimmed milk powders obtained by Pulse Spray Drying (PSD) and Spray Drying (SD). BI: browning index, S_I: solubility index, H_R: Hausner ratio; [α-La]: α-Lactalbumin, [β-Lg]: β-Lactoglobulin. *(n=2); +(n=4)

T (°C)	DT	[α-La] (g/Kg)	[β-Lg] (g/Kg)	Bulk density (g/mL)	Compressibility (Carr Index) (%)	H_R	BI	D_{10} (μm)	D_{50} (μm)	D_{90} (μm)	Span	$D_{[3.2]}$ (μm)	$D_{[4.3]}$ (μm)	L*	a*	b*	Moisture (%)	Flowability (g/min)	Solubility Index (%)	Protein (%)
70	PSD	9.65*	40.04	0.51	35.71	1.56	15.49	13.90	48.00	147.00	2.77	30.60	69.30	58.67	-1.99	10.00	3.08	18.59	98.60	33.16
		12.23	54.49	0.53	34.74	1.53	16.53	12.05	39.75	104.15	2.26	26.05	50.73	58.50	-2.30	10.70	3.48	23.87	98.61	33.49
		**10.94 ± 1.83+**	**47.27 ± 10.22**	**0.52 ± 0.01**	**35.23 ± 0.69**	**1.54 ± 0.02**	**16.01 ± 0.57**	**12.98 ± 1.31**	**43.88 ± 5.83**	**125.58 ± 30.30**	**2.52 ± 0.36**	**28.33 ± 3.22**	**60.01 ± 13.14**	**58.58 ± 0.08**	**-2.15 ± 0.16**	**10.35 ± 0.35**	**3.28 ± 0.28**	**21.23 ± 3.74**	**98.61 ± 0.01**	**33.32 ± 0.24**
	SD	10.38	49.12	0.47	36.54	1.58	12.35	19.85	55.75	272.50	4.51	41.95	103.50	61.62	-2.07	8.79	4.02	11.02	99.53	34.35
		13.38	61.00	0.56	32.22	1.48	13.61	19.10	55.80	272.00	4.55	41.90	104.00	60.52	-2.57	9.66	2.28	8.80	99.72	34.08
		**11.88 ± 2.12**	**55.06 ± 8.40**	**0.52 ± 0.06**	**34.38 ± 3.05**	**1.53 ± 0.07**	**12.98 ± 0.69**	**19.48 ± 0.53**	**55.78 ± 0.04**	**272.25 ± 0.35**	**4.53 ± 0.03**	**41.93 ± 0.04**	**103.75 ± 0.35**	**61.07 ± 0.55**	**-2.32 ± 0.25**	**9.23 ± 0.44**	**3.15 ± 1.23**	**9.91 ± 1.57**	**99.63 ± 0.14**	**34.22 ± 0.19**
80	PSD	10.54	41.87	0.50	32.00	1.47	14.47	15.50	49.00	108.00	1.89	31.90	56.90	59.36	-2.07	9.63	2.77	35.95	98.51	35.37
		9.98	45.10	0.50	33.00	1.99	14.48	15.45	49.25	111.50	1.95	32.05	58.00	61.31	-2.09	9.92	4.11	27.59	98.13	35.46
		**10.26 ± 0.39**	**43.48 ± 2.29**	**0.50 ± 0.01**	**32.50 ± 0.71**	**1.48 ± 0.02**	**14.48 ± 0.01**	**15.48 ± 0.04**	**49.13 ± 0.18**	**109.75 ± 2.48**	**1.92 ± 0.04**	**31.98 ± 0.12**	**57.45 ± 0.78**	**60.34 ± 0.98**	**-2.08 ± 0.01**	**9.78 ± 0.14**	**3.44 ± 0.95**	**31.77 ± 5.91**	**98.32 ± 0.27**	**35.42 ± 0.06**
	SD	11.91	44.24	0.55	27.47	1.38	11.32	9.13	32.80	86.70	2.36	19.60	51.10	58.33	-2.02	7.82	2.15	17.24	99.45	34.64
		10.36	49.34	0.51	34.69	1.53	11.19	8.93	32.50	86.10	2.37	19.30	50.60	63.88	-2.30	8.55	4.66	4.82	99.68	34.34
		**11.14 ± 1.09**	**46.79 ± 3.61**	**0.53 ± 0.03**	**31.08 ± 5.11**	**1.46 ± 0.11**	**11.25 ± 0.07**	**9.03 ± 0.14**	**32.65 ± 0.21**	**86.40 ± 0.42**	**2.37 ± 0.01**	**19.45 ± 0.21**	**50.85 ± 0.35**	**61.11 ± 2.78**	**-2.16 ± 0.14**	**8.19 ± 0.36**	**3.40 ± 1.77**	**11.03 ± 8.78**	**99.57 ± 0.16**	**34.49 ± 0.21**
90	PSD	11.27	39.32	0.43	36.21	1.57	15.82	14.60	43.70	91.40	1.90	29.20	49.20	58.65	-2.00	10.17	2.34	16.87	98.38	34.06
		9.33	43.13	0.47	33.96	1.51	14.70	14.65	43.65	91.40	1.76	29.20	49.15	60.16	-2.06	9.86	1.59	19.17	98.16	34.10
		**10.30 ± 1.38**	**41.23 ± 2.70**	**0.45 ± 0.03**	**35.08 ± 1.59**	**1.54 ± 0.04**	**15.26 ± 0.61**	**14.63 ± 0.04**	**43.68 ± 0.04**	**91.40 ± 0.01**	**1.83 ± 0.10**	**29.20 ± 0.01**	**49.18 ± 0.04**	**59.41 ± 075**	**-2.03 ± 0.03**	**10.01 ± 0.16**	**1.96 ± 0.53**	**18.02 ± 1.62**	**98.27 ± 0.16**	**34.08 ± 0.03**
	SD	12.11	41.82	0.51	32.65	1.48	15.65	9.89	38.00	109.00	2.61	21.50	59.50	59.19	-2.55	10.52	3.12	10.70	99.36	35.86
		9.98	46.49	0.47	39.25	1.65	11.79	9.47	37.40	102.00	2.47	20.90	54.50	58.64	-2.00	8.08	2.32	13.65	99.19	35.58
		**11.04 ± 1.50**	**44.15 ± 3.30**	**0.49 ± 0.03**	**35.95 ± 4.67**	**1.57 ± 0.11**	**13.71 ± 2.13**	**9.68 ± 0.30**	**37.70 ± 0.42**	**105.50 ± 4.95**	**2.54 ± 0.09**	**21.20 ± 0.42**	**57.00 ± 3.54**	**58.92 ± 0.28**	**-2.27 ± 0.27**	**9.30 ± 1.22**	**2.72 ± 0.57**	**12.18 ± 2.09**	**99.27 ± 0.13**	**35.72 ± 0.19**
100	PSD	11.64	51.51	0.40	37.90	1.61	14.51	12.80	37.30	75.80	1.69	24.70	41.40	59.79	-1.81	9.54	1.00	9.39	98.59	36.20
		8.30	42.83	0.48	28.85	1.41	14.73	12.60	37.20	75.70	1.70	24.20	41.30	62.71	-2.06	10.23	0.63	5.01	98.20	35.79
		**9.97 ± 2.36**	**47.11 ± 6.14**	**0.44 ± 0.05**	**33.37 ± 6.40**	**1.51 ± 0.15**	**14.62 ± 0.12**	**12.70 ± 0.14**	**37.25 ± 0.07**	**75.75 ± 0.07**	**1.69 ± 0.01**	**24.45 ± 0.35**	**41.35 ± 0.07**	**61.25 ± 1.46**	**-1.94 ± 0.13**	**9.89 ± 0.35**	**0.82 ± 0.26**	**7.20 ± 3.10**	**98.39 ± 0.28**	**35.99 ± 0.28**
	SD	10.29	54.79	0.44	30.09	1.43	14.30	26.00	57.90	145.00	2.06	43.50	79.00	59.59	-2.38	9.78	2.57	16.11	99.66	35.86
		10.04	51.52	0.40	36.51	1.58	12.36	25.95	57.90	145.00	2.06	43.55	79.05	61.95	-2.22	8.94	2.53	3.08	99.50	35.06
		**10.16 ± 0.18**	**53.15 ± 2.32**	**0.42 ± 0.03**	**33.30 ± 4.54**	**1.50 ± 0.10**	**13.33 ± 1.06**	**25.98 ± 0.04**	**57.90 ± 0.01**	**145.00 ± 0.01**	**2.06 ± 0.01**	**43.53 ± 0.04**	**79.03 ± 0.04**	**60.77 ± 1.18**	**-2.30 ± 0.08**	**9.36 ± 0.42**	**2.55 ± 0.03**	**9.60 ± 9.21**	**99.58 ± 0.11**	**35.46 ± 0.56**

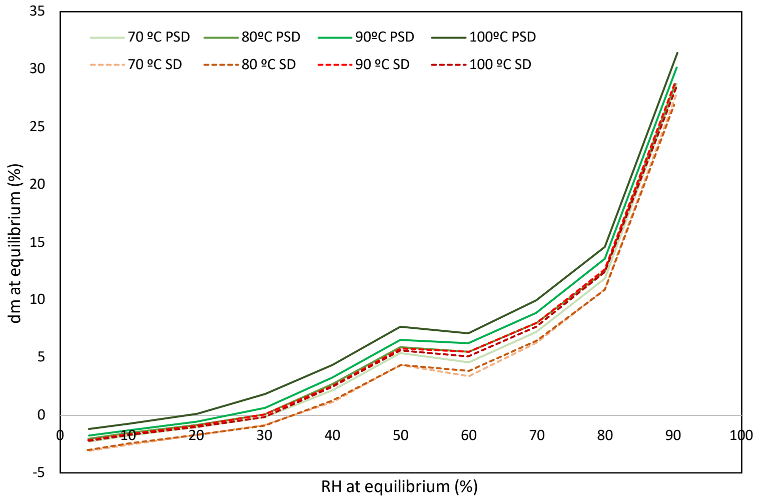

Figure S1. Mass increment (%) versus the relative humidity of SMP obtained by Spray Drying (SD) and pulse Spray Drying (PSD) at different outlet temperatures (70, 80, 90 and 100 °C).
